# Left atrial appendage flow velocity predicts recurrence of atrial fibrillation after catheter ablation: A systematic review and meta-analysis

**DOI:** 10.3389/fcvm.2022.971848

**Published:** 2022-09-06

**Authors:** Pengfei Chen, Yujiao Shi, Jianqing Ju, Deng Pan, Lina Miao, Xiaolin Guo, Zhuhong Chen, Jianpeng Du

**Affiliations:** ^1^Xiyuan Hospital, China Academy of Chinese Medical Sciences, Beijing, China; ^2^Cardiovascular Diseases Center, Xiyuan Hospital, China Academy of Chinese Medical Sciences, Beijing, China; ^3^Beijing University of Traditional Chinese Medicine, Beijing, China; ^4^Shanxi University of Traditional Chinese Medicine, Taiyuan, China

**Keywords:** left atrial appendage flow velocity, atrial fibrillation, catheter ablation, recurrence, systematic review, meta-analysis

## Abstract

**Purpose:**

There is increasing evidence that left atrial appendage flow velocity (LAAFV) is linked to the recurrence of atrial fibrillation (AF) after catheter ablation (CA), suggesting the potential predictable significance of LAAFV in this setting. We performed a systematic review and meta-analysis to assess whether LAAFV is association with AF recurrence after CA.

**Methods:**

Up to May 1, 2022, six databases (PubMed, EMBASE, Web of Science, Cochrane Library, Scopus, and CINAHL) were searched for literature reporting the association between LAAFV and AF recurrence after CA. All statistical analyses were carried out using STATA version 16 software. Heterogeneity was determined by the Cochrane’s Q test and I^2^ statistics. The Newcastle-Ottawa Scale (NOS) was used to assess the methodological quality of each included study, and the Grading of Recommendations Assessment, Development and Evaluation (GRADE) method was adopted to evaluate the quality of evidence.

**Result:**

Sixteen studies with 5,006 AF patients after CA (1,479 patients with AF recurrence, 3,527 without AF recurrence) were included in the meta-analysis. The meta-analysis of 15 studies (16 data sets) showed that patients with recurrence exhibited lower LAAFV values than those without recurrence [standardized mean difference (SMD): −0.65, 95% CI: −0.88 to −0.42, *P* < 0.01]. Moreover, we evaluated the association of LAAFV and the risk of AF recurrence after CA. Nine studies (11 data sets) defined LAAFV as continuous variables, and the pooled analysis suggested that for every 1 cm/s rise in LAAFV values, the risk of AF recurrence after CA decreased by 3% [Odds Ratio (OR): 0.97, 95% CI: 0.95 to 0.99, *P* < 0.01]. Seven studies defined LAAFV as categorical variables, and the pooled analysis showed that lower LAAFV were associated with an increased risk of AF recurrence after CA [OR: 2.28, 95% CI: 1.46 to 3.57, *P* < 0.01]. The subgroup analyses showed that the association between LAAFV and AF recurrence after CA was not significantly affected by the AF type and ablation procedure. The NOS indicated that included studies were moderate to high quality, while the GRADE assessment suggested a low certainty of the evidence.

**Conclusion:**

Lower LAAFV may be associated with an increased risk of AF recurrence after CA. Further studies with well designed and randomized studies for LAAFV should be conducted.

**Systematic review registration:**

[https://www.crd.york.ac.uk/PROSPERO/], identifier [CRD42022333627].

## Introduction

Atrial fibrillation (AF), affecting more than 46.3 million individuals worldwide, is the most common sustained arrhythmia (1). As one of the main risk factors for heart failure, myocardial infarction, and thromboembolic strokes, AF is associated with high mortality and hospitalization rates and ultimately imposes a considerable burden on individuals and society (2). Catheter ablation (CA), an effective therapeutic option for drug-refractory symptomatic AF (3), was recommended as first-line therapy for patients with paroxysmal AF in a recent meta-analysis (4). However, AF recurrence remains a challenging issue with a recurrence rate of up to 30% (5), which make it more essential to use screening factors to predict patients at high risk of AF recurrence and post-operative complications. The risk factors for AF recurrence include age, type of AF, duration of AF, left atrial (LA) enlargement, left ventricular ejection fraction, structure and function of left atrial appendage (LAA), atrial natriuretic peptide level, sleep apnea, obesity, and hypertension (6–8). The investigations of the association between LAAFV and AF recurrence following CA have increased exponentially in recent years. Nevertheless, these studies were small and contradictory. Therefore, we performed a systematic review and meta-analysis to evaluate whether LAAFV is association with AF recurrence after CA.

## Methods

This systematic review and meta-analysis was reported followed the criteria outlined in the Meta-Analysis of Observational Studies in Epidemiology (MOOSE) and the PRISMA 2020 (9). The systematic evaluation program for this study was registered in PROSPERO (number: CRD42022333627).

### Search strategy

A systematic literature search was conducted independently by two investigators (Peng-fei Chen and Yu-jiao Shi) in six databases (PubMed, EMBASE, Web of Science, Cochrane Library, Scopus, and CINAHL). We searched for English-language literature published up to May 1, 2022. The following search MESH terms and keywords were used: “left atrial appendage flow velocity,” “atrial fibrillation,” “catheter ablation,” “radiofrequency ablation,” “cryoablation,” “recurrence.” Detailed search strategies were shown in the Supplementary material. The disagreements were resolved by consulting a third investigator (Jian-peng Du).

### Eligible studies

Two investigators (Peng-fei Chen and Yu-jiao Shi) independently screened titles, abstracts, and full-text material to select eligible studies. The criteria for inclusion in this meta-analysis were as follows: (1) observational studies with at least 6 months and completeness of follow-up period; (2) the target population was patients with AF (including paroxysmal AF and persistent AF) after CA (including radiofrequency and cryoballoon ablation); (3) comparing means and standard deviations (SDs) of LAAFV values in individuals with AF recurrence after CA to those without recurrence; and (4) reporting odds ratios (ORs) or hazard ratios (HRs) and the corresponding 95% confidence intervals (CIs) for LAAFV as a predictor of AF recurrence after CA. The abstracts, editorial, animal experiment, or review were excluded.

### Data extraction

Pre-specified data variables were extracted independently by two investigators (Peng-fei Chen and Yu-jiao Shi) using a standard form. The following information was extracted from the eligible literature: the first author’s name, publication year, study location, study design, baseline characteristics of patients (gender, age, and sample size), numbers of patients (paroxysmal AF patients, persistent AF patients, and recurrent post-operative patients), LAAFV measurement method, blanking period in months, and follow-up duration in months.

### Quality evaluation

The quality of the included research was assessed according to the Newcastle-Ottawa Scale (NOS), an assessment tool focused on three aspects: participant selection, comparability, and exposure. The NOS score ranged from 0 to 9, with more than or equal to 8 stars defined as high quality, 6–8 stars as moderate quality, and less than 6 stars as low quality (10). To assess the certainty of the evidence, we adopted the Grading of Recommendations Assessment, Development and Evaluation (GRADE) approach (11), which classifies evidence as high, moderate, low, or very low certainty based on the following factors: risk of bias, inconsistency, indirectness, imprecision, and publication bias. Since all included studies were observational studies, the preliminary definition of the quality evidence was low, and other factors may then upgrade or downgrade the quality level. Any disagreements were resolved by consulting a third investigator (Jian-peng Du).

### Statistical analysis

In the analysis of LAAFV values in AF patients with and without recurrence after CA, means and SDs of LAAFV values were extracted, and standardized mean difference (SMD) and 95% CI were calculated for each study. In analyzing the association between LAAFV and the risk of AF recurrence after CA, univariate or multivariate ORs for AF recurrence reported by logistic regression analysis were extracted. For the study that only reported HRs, HRs was adopted as the best estimate of ORs. Since the studies included in this meta-analysis used LAAFV values as either a categorical or continuous variable to evaluate ORs, two separate meta-analyses were conducted for both variables. Heterogeneity was assessed by the Cochrane’s Q test (*P* < 0.1 was considered statistical heterogeneity) and I^2^ Statistics (25, 50, and 75% were considered to represent low, medium, and high heterogeneity, respectively). We adopted a random-effect model for the meta-analysis because it incorporates the potential effects of heterogeneity and therefore allows for the retrieval of more generalizable results. Subgroup analyses were stratified by study location (Europe or Asia), study design (prospective or retrospective), sample size (≤100 or >100), AF type (persistent AF or paroxysmal AF), ablation procedure (circumferential pulmonary vein isolation or additional linear ablations), follow-up time (≤12 or >12 months), and ablation type (cryoballoon ablation or radiofrequency ablation). The inverted funnel plot and Egger’s test were performed to assess publication bias. Sensitivity analyses by removing one individual study at a time to confirm the robustness of the results. All statistical analyses were carried out using STATA version 16 software.

## Results

### Study search

**FIGURE 1:**
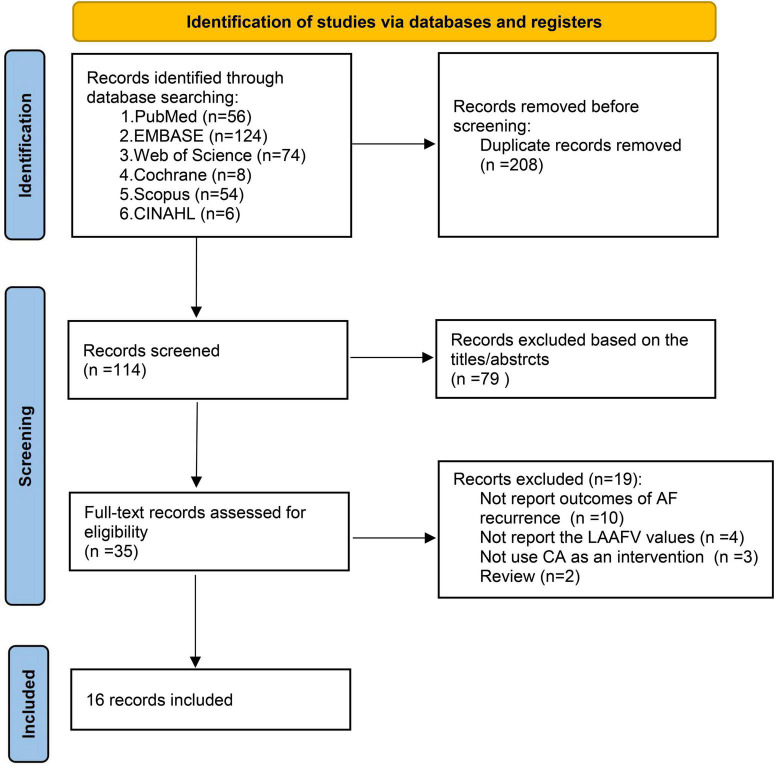
Flow diagram of study selection and identification.

The database search and study identification procedure was presented in Figure 1. A total of 322 records were retrieved, 208 of which were duplicates, and 79 studies were excluded after reading the title and abstract, primarily because they were irrelevant to the study purpose. The remaining 35 articles were evaluated for eligibility by full-text screening. Of these, 19 studies were further excluded because 10 did not report the AF recurrence outcomes, 4 did not report the LAAFV values, 3 did not use CA as an intervention, and the other 2 were reviews. Finally, 16 studies (12–27) were included in our systematic review and meta-analysis.

### Study characteristics

Table 1 displayed the study characteristics of 16 included studies, comprising 9 (12, 15, 18, 20, 21, 23–25, 27) prospective studies and 7 (13, 14, 16, 17, 19, 22, 26) retrospective studies. Six studies (19, 22–25, 27) were conducted in China, 4 studies (15, 16, 20, 21) in Japan, 2 studies (14, 17) in Hungary, 1 study (26) in South Korea, 1 study (13) in Poland, 1 study (12) in Türkiye, and 1 study (18) in Romania. Sixteen studies with 5,006 AF patients after CA (1,479 patients with AF recurrence, 3,527 without AF recurrence) were included, and the proportion of AF patients with recurrence ranged from 24.1 to 41.5%. The proportion of males was higher than that of females, varying from 59.3 to 92.7%. The mean age ranged from 54.6 ± 10.4 to 67.5 ± 7.5 years, and the median follow-up time varied from 6 to 48 months. Nine (12–15, 17, 18, 22, 23, 25) studies performed circumferential pulmonary vein isolation (CPVI) alone, whereas 7 (16, 19–21, 24, 26, 27) studies conducted additional linear ablations. The LAAFV, which refers to the peak flow emptying velocity of the left atrial appendage at late diastole, was measured by transthoracic echocardiography (TEE) in all selected research. All studies used 24-h Holter and/or surface electrocardiogram recording to diagnose asymptomatic AF recurrence. All studies blanking period of post-CA procedure were 3 months, except for 1 study (15) was 2 months.

**TABLE 1:** Characteristics of 16 studies included in the meta-analysis of difference in LAAFV between patients with and without post-CA AF recurrence.

References	Study location	Study design	No. of patients	Patients with recurrence	Age (years)	Men (%)	Paroxysmal AF (%)
Gerede et al. (12)	Türkiye	prospective	51	16(31.3%)	54.6 ± 10.4	25(49%)	51
Kiełbasa et al. (13)	Poland	retrospective	417	107(25.7%)	59	253(60.3%)	417
Simon et al. (14)	Hungary	retrospective	561	229(40.8%)	61.9 ± 10.2	365(65.1%)	376
Fukushima et al. (15)	Japan	prospective	105	39(37.1%)	57 ± 12	86 (73.5%)	105
Ariyama et al. (16)	Japan	retrospective	41	17(41%)	58 ± 10	38(93%)	0
Szegedi et al. (17)	Hungary	retrospective	428	143(33.4%)	60.7 ± 10.8	276(64.5%)	143
Istratoaie et al. (18)	Romania	prospective	81	24(29.6%)	55.3 ± 9	48(59.3%)	81
Gong et al. (19)	China	retrospective	84	22(26.2%)	67.5 ± 7.5 66.1 ± 9.0	58(69%)	60
Kanda et al. (20)	Japan	prospective	53	16(30%)	65 ± 10	42(79%)	0
Shiozawa et al. (21)	Japan	prospective	77	28(36%)	59 ± 8	62(81%)	49
Yang et al. (22)	China	retrospective	164	43(26.2%)	58.2 ± 9.7	126(76.8%)	0
Ma et al. (23)	China	prospective	120	39(32.5%)	64 ± 7	72(60%)	55
Ma et al. (24)	China	prospective	124	41(33.1%)	65.5 ± 6.0 62.6 ± 7.3	75(60.5%)	58
He et al. (25)	China	prospective	80	24(30%)	57.31 ± 10.42	48(60%)	80
Kim et al. (26)	Korea	retrospective	2352	613(26.1)%)	55.4 ± 10.9	1872(79.6%)	1401
Yang et al. (27)	China	prospective	228	55(24.1%)	62.9 ± 9.4 62.8 ± 9.6	188(82.5%)	0

**References**	**Ablation procedure**	**Ablation types**	**Measurement of asymptomatic** **recurrence**	**Blanking period** **(months)**	**Follow-up** **(months)**	**image used**	**NOS**

Gerede et al. (12)	CPVI	CYA	surface ECG, 24-h Holter recording, and clinical assessment.	3 m	12 m	TEE	8
Kiełbasa et al. (13)	CPVI	CYA	surface ECG, 24-h Holter recording and intracardiac electrogram from the implanted device	3 m	24 m	TEE	8
Simon et al. (14)	CPVI	RFCA	surface ECG, 24-h Holter recording, and clinical assessment	3 m	12 m	TEE	7
Fukushima et al. (15)	CPVI	RFCA	surface ECG and 24-h Holter recording	2 m	12 m	TEE	8
Ariyama et al. (16)	CPVI plus	RFCA	surface ECG and 24-h Holter recording	3 m	12 m	TEE	7
Szegedi et al. (17)	CPVI	RFCA	24-h Holter recording, and clinical assessment	3 m	43 m	TEE	7
Istratoaie et al. (18)	CPVI	RFCA	surface ECG and 24-h Holter recording	3 m	12 m	TEE	8
Gong et al. (19)	CPVI plus	RFCA	12-lead surface ECG and 24-h Holter recording	3 m	48 m	TEE	7
Kanda et al. (20)	CPVI plus	RFCA	12-lead surface ECG, 24-h Holter recording, and clinical assessment	3 m	12 m	TEE	9
Shiozawa et al. (21)	CPVI plus	RFCA	12-lead surface ECG and 24-h Holter recording	3 m	12 m	TEE	7
Yang et al. (22)	CPVI	RFCA	12-lead surface ECG and 24-h Holter recording	3 m	24 m	TEE	9
Ma et al. (23)	CPVI	RFCA	12-lead surface ECG and 24-h Holter recording	3m	12 m	TEE	8
Ma et al. (24)	CPVI plus	RFCA	12-lead surface ECG and 24-h Holter recording	3 m	12 m	TEE	8
He et al. (25)	CPVI	RFCA	12-lead surface ECG and 24-h Holter recording	3 m	12 m	TEE	7
Kim et al. (26)	CPVI plus	RFCA	12-lead surface ECG and 24-h Holter recording	3 m	12 m	TEE	7
Yang et al. (27)	CPVI plus	RFCA	12-lead surface ECG and 24-h Holter recording	3 m	6 m	TEE	6

For complete study names, see Reference. CPVI, circumferential pulmonary vein isolation; CPVI plus, includes CPVI with one or more of adjuvant ablations in cavotricuspid isthmus, mitral isthmus, left atrial roof, the basal posterior wall, superior vena cava or complex fractionate atrial electrograms; RFCA, radiofrequency ablation; CYA, cryoballoon ablation; ECG, electrocardiogram; TEE, transoesophageal echocardiography.

### Study quality

Based on NOS for observational studies, all examined studies had quality ratings ranging from 6 to 9 (mean score: 7.6), indicating moderate to high quality. Detailed quality assessment is presented in Table 2.

**TABLE 2:** Quality assessment of the 16 included studies was assessed by the Newcastle–Ottawa scale.

Study (First,Author, Year)	Select				Comparability	Outcome			Total
	Exposed cohort	Non-exposed cohort	Ascertainment of exposure	Outcome of interest		Assessment of outcome	Length of follow-up	Adequacy of follow-up	
Gerede et al. (12)	*	*	*	*	*	*	*	*	8
Kiełbasa et al. (13)	*	*	*	*	*	*	*	*	8
Simon et al. (14)	*	*	*		*	*	*	*	7
Fukushima et al. (15)	*	*	*	*	*	*	*	*	8
Ariyama et al. (16)	*	*	*		*	*	*	*	7
Szegedi et al. (17)	*	*	*		*	*	*	*	7
Istratoaie et al. (18)	*	*	*	*	*	*	*	*	8
Gong et al. (19)	*	*	*		*	*	*	*	7
Kanda et al. (20)	*	*	*	*	**	*	*	*	9
Shiozawa et al. (21)	*	*	*		*	*	*	*	7
Yang et al. (22)	*	*	*	*	**	*	*	*	9
Ma et al. (23)	*	*	*	*	*	*	*	*	8
Ma et al. (24)	*	*	*	*	*	*	*	*	8
He et al. (25)	*	*	*		*	*	*	*	7
Kim et al. (26)	*	*	*		*	*	*	*	7
Yang et al. (27)	*	*	*	*	*	*			6

*Represents one point, **represents two points in the Newcastle-Ottawa Scale.

According to the GRADE grade system, the evidence supporting the link between LAAFV and AF recurrence following CA was of low certainty. Table 3 displays the certainty assessment ratings and a description of the results.

**TABLE 3:** AF recurrence outcomes and GRADE classification in meta-analysis of observational studies.

No of studies	Certainty assessment						Effect			Certainty	Importance
	Study design	Risk of bias	Inconsistency	Indirectness	Imprecision	Other considerations	No of recurrence	No of not recurrence	Relative (95% CI)		
The difference in LAAFV values between patients with and without AF recurrence after CA											
15	observational studies	not serious	very serious*^a^*	not serious	not serious	strong association*^b^* all plausible residual confounding would reduce the demonstrated effect*^c^*	1349	3200	SMD −0.65 (−0.88 to −0.42)	⊕⊕°° Low	crucial
The risk of AF recurrence after CA for the increment of LAAFV values of 1 cm/s											
9	observational studies	not serious	very serious*^a^*	not serious	not serious	all plausible residual confounding would reduce the demonstrated effect*^c^* dose response gradient*^d^*	1163	2807	OR 0.97 (0.95 to 0.99)	⊕⊕°° Low	crucial
The risk of AF recurrence after CA for patients with higher versus lower LAAFV values											
7	observational studies	not serious	very serious*^a^*	not serious	not serious	strong association*^b^* all plausible residual confounding would reduce the demonstrated effect*^c^*	858	2365	OR 2.28 (1.46 to 3.57)	⊕⊕°° Low	crucial

a. The score was downgraded because substantial heterogeneity between studies was detected and could not be fully explained downgraded.

b. The score was upgraded because the magnitude of the effect was large (SMD < −0.5 and OR > 2) upgraded.

c. The score was downgraded because all included studies in this meta-analysis were observational studies, we cannot rule out that some residual factors may reduce the demonstrated effect downgraded.

d. The score was upgraded because there was evidence of significant dose–response association (every 1 cm/s rise in LAAFV values, the risk of AF recurrence decreased by 3%) upgraded.

CI confidence interval, OR odds ratio, SMD standardized mean difference, AF atrial fibrillation, CA catheter ablation, LAAFV left atrial appendage flow velocity.

### Results of meta-analysis

#### The difference in left atrial appendage flow velocity values between patients with and without atrial fibrillation recurrence after catheter ablation

**FIGURE 2:**
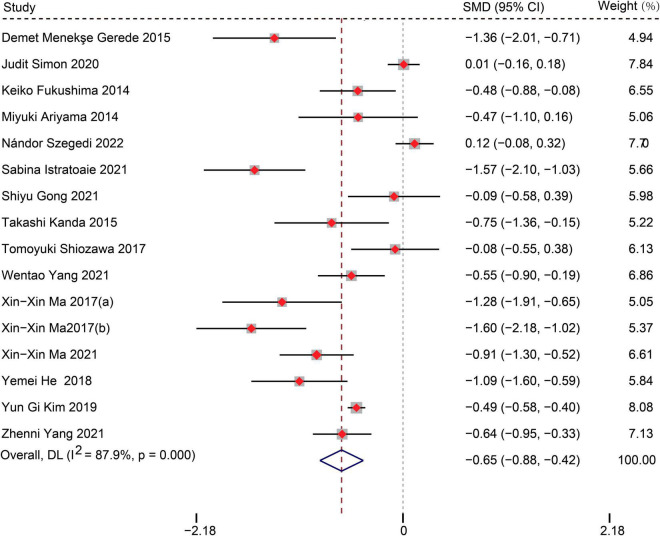
Forest plots show the difference in LAAFV values between patients with and without AF recurrence after CA.

Fifteen studies (12, 14–27) (16 data sets) reported the difference in LAAFV values between patients with (*n* = 1,349) and without (*n* = 3,200) AF recurrence after CA. Patients with recurrence exhibited lower LAAFV values than those without recurrence [SMD:−0.65, 95% CI: −0.88 to −0.42, *P* < 0.01; *I^2^* = 87.9% Figure 2]. The Funnel plot was symmetrical upon visual inspection (Supplementary Figure 1), and the *P*-value of Egger’s test was 0.12 (Supplementary Figure 2), indicating no significant publication bias. The sensitivity analysis results were consistent (SMD: −0.71 to −0.59, *p* all < 0.05, Supplementary Figure 3). The subgroup analyses were summarized in Table 4. When we performed subgroup analyses stratified by study design and follow-up time, the results were not statistically significant in the subgroup of retrospective study [SMD: −0.23, 95% CI: −0.52 to 0.06, *P* > 0.05] and follow-up times > 12m study [SMD: −0.16, 95% CI: −0.60 to 0.28, *P* > 0.05].

**TABLE 4:** Subgroup analyses of difference in LAAFV values between patients with and without AF recurrence after CA.

Subgroup	Study	Number of study	Meta-analysis			Heterogeneity	
			Effect size	95%CI	*P*-value	I^2^	*P*-value
Study location	Europe	4	−0.62	−1.21 to −0.02	*p* < 0.05	93.9%	*p* < 0.01
	Asia	12	−0.66	−0.86 to −0.47	*p* < 0.01	68.7%	*p* < 0.01
Study design	Prospective	10	−0.94	−1.24 to −0.65	*p* < 0.01	73.6%	p < 0.01
	Retrospective	6	−0.23	−0.52 to 0.06	*p* > 0.05	89.7%	*p* < 0.01
Sample size	Numbers ≤ 100	7	−0.76	−1.22 to −0.30	*p* < 0.01	79.7%	*p* < 0.01
	Numbers > 100	9	−0.57	−0.85 to −0.30	*p* < 0.01	90.7%	*p* < 0.01
AF type	Persistent AF	5	−0.77	−1.11 to −0.43	*p* < 0.01	62.4%	*p* < 0.05
	Paroxysmal AF	6	−0.95	−1.43 to −0.48	*p* < 0.01	79.5%	*p* < 0.01
Ablation procedure	CPVI	9	−0.82	−1.24 to −0.39	*p* < 0.01	92.1%	*p* < 0.01
	CPVI plus	7	−0.51	−0.70 to −0.32	*p* < 0.01	48.1%	*p* > 0.05
Follow-up time	Times > 12 m	3	−0.16	−0.60 to 0.28	*p* > 0.05	80.8%	*p* < 0.01
	Times ≤ 12 m	13	−0.77	−1.03 to −0.52	*p* < 0.01	86.7%	*p* < 0.01

CPVI, circumferential pulmonary vein isolation; CPVI plus, includes CPVI with one or more of adjuvant ablations in cavotricuspid isthmus, mitral isthmus, left atrial roof, the basal posterior wall, superior vena cava or complex fractionate atrial electrograms; AF, atrial fibrillation.

#### The risk of atrial fibrillation recurrence after catheter ablation for the increment of left atrial appendage flow velocity values of 1 cm/s

**FIGURE 3:**
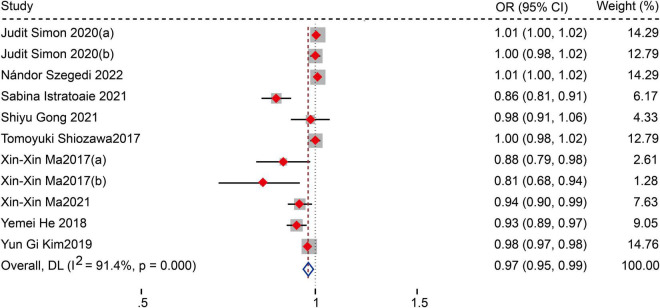
Forest plots show the relationship between LAAFV (continuous variables) and the risk of AF recurrence after CA.

Fourteen studies (12–15, 17–27) reported the relationship between LAAFV values and the risk of AF recurrence after CA. Nine studies (14, 17–19, 21, 23–26) (11 data sets) defined LAAFV as continuous variables, and the pooled analysis showed that for every 1cm/s rise in LAAFV values, the risk of AF recurrence after CA decreased by 3% [OR:0.97, 95% CI: 0.95 to 0.99, *P* < 0.01; *I*^2^ = 91.4% Figure 3]. The sensitivity analysis results were consistent (OR: 0.96 to 0.98, *p* all < 0.05, Supplementary Figure 4). The subgroup analyses were summarized in Table 5, and the effect sizes were consistent regardless of AF types and ablation procedure. When we stratified the studies by study location, study design, sample size, and follow-up time, the results were not statistically significant in the subgroup of Europe study [OR: 0.99, 95% CI: 0.96 to 1.01, *P* > 0.05], retrospective study [OR: 1, 95% CI: 0.98 to 1.02, *P* > 0.05], numbers < 100 study [OR: 0.94, 95% CI: 0.88 to 1.01, *P* > 0.05], numbers > 100 study [OR: 0.98, 95% CI: 0.96 to 1.01, *P* > 0.05], and follow-up times > 12 m [OR: 1.01, 95% CI: 1.00 to 1.02, *P* > 0.05].

**TABLE 5:** Subgroup analyses of the risk of AF recurrence after CA based on LAAFV (continuous variable).

Subgroup	Study	Number of study	Meta-analysis			Heterogeneity	
			Effect size	95%CI	*P*-value	I^2^	*P*-value
Study location	Europe	4	0.99	0.96–1.01	*p* > 0.05	90.10%	*p* < 0.01
	Asia	7	0.96	0.94–0.98	*p* < 0.01	73.70%	*p* < 0.01
Study design	Prospective	6	0.91	0.86–0.97	*p* < 0.01	87.60%	*p* < 0.01
	Retrospective	5	1	0.98–1.02	*p* > 0.05	94.30%	*p* < 0.01
Sample size	Numbers ≤ 100	4	0.94	0.88–1.01	*p* > 0.05	90.30%	*p* < 0.01
	Numbers > 100	7	0.98	0.96–1.01	*p* > 0.05	92.90%	*p* < 0.01
AF type	Persistent AF	1	0.81	0.69–0.95	*p* < 0.05	0%	–
	Paroxysmal AF	4	0.91	0.86–0.96	*p* < 0.01	66.30%	*p* < 0.05
Ablation procedure	CPVI	7	0.96	0.94–0.99	*p* < 0.01	89.70%	*p* < 0.01
	CPVI plus	4	0.98	0.96–1.00	*p* < 0.05	61.90%	*p* < 0.01
Follow-up time	Times > 12m	2	1.01	1.00–1.02	*p* > 0.05	0%	*p* < 0.05
	Times ≤ 12m	9	0.96	0.94–0.99	*p* < 0.01	90.80%	*p* < 0.01

CPVI, circumferential pulmonary vein isolation; CPVI plus, includes CPVI with one or more of adjuvant ablations in cavotricuspid isthmus, mitral isthmus, left atrial roof, the basal posterior wall, superior vena cava or complex fractionate atrial electrograms; AF, atrial fibrillation.

#### The risk of atrial fibrillation recurrence after catheter ablation for patients with higher versus lower left atrial appendage flow velocity values

**FIGURE 4:**
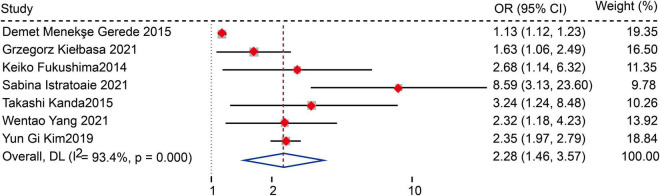
Forest plots show the relationship between LAAFV (categorical variables) and the risk of AF recurrence after CA.

Seven studies (12, 13, 15, 18, 20, 22, 26) defined LAAFV as categorical variables. The pooled analysis showed that lower LAAFV was associated with an increased risk of AF recurrence after CA [OR:2.28, 95% CI: 1.46 to 3.57, *P* < 0.01; *I*^2^ = 93.4% Figure 4]. The sensitivity analysis results were consistent (OR: 1.96 to 2.48, *p* all < 0.05, Supplementary Figure 5). The subgroup analyses were summarized in Table 6. When we stratified the studies by study location, sample size, and ablation type, the results were not statistically significant in the subgroup of Europe study [OR: 2.05, 95% CI: 0.99 to 4.22, *P* > 0.05], numbers < 100 study [OR: 2.93, 95% CI: 0.81 to 10.65, *P* > 0.05], and cryoballoon ablation study [OR: 1.27, 95% CI: 0.97 to 1.78, *P* > 0.05].

**TABLE 6:** Subgroup analyses of the risk of AF recurrence after CA based on LAAFV (categorical variable).

Subgroup	Study	Number of study	Meta-analysis			Heterogeneity	
			Effect size	95% CI	*P*-value	I^2^	*P*-value
Study location	Europe	3	2.05	0.99–4.22	*p* > 0.05	89.00%	*p* < 0.01
	Asia	4	2.38	2.02–2.80	*p* < 0.01	0%	*p* > 0.05
Study design	Prospective	4	2.81	1.08–7.33	*p* < 0.05	87.45%	*p* < 0.01
	Retrospective	3	2.18	1.77–2.68	*p* < 0.01	17.80%	*p* > 0.05
Sample size	Numbers ≤ 100	3	2.93	0.81–10.65	*p* > 0.05	90%	*p* < 0.01
	Numbers > 100	4	2.25	1.93–2.62	*p* < 0.01	0%	*p* > 0.05
AF type	Persistent AF	2	2.57	1.51–4.38	*p* < 0.01	0%	*p* > 0.05
	Paroxysmal AF	4	2.16	1.14–4.08	*p* < 0.05	86.40%	*p* < 0.01
Ablation procedure	CPVI	5	2.17	1.25–3.76	*p* < 0.01	85%	*p* < 0.01
	CPVI plus	2	2.37	2.00–2.82	*p* < 0.01	0%	*p* > 0.05
Follow-up time	Times > 12m	2	1.82	1.27–2.59	*p* < 0.01	0%	*p* > 0.05
	Times ≤ 12m	5	2.50	1.41–4.43	*p* < 0.01	95.30%	*p* < 0.01
Ablation type	CYA	2	1.27	0.97–1.78	*p* > 0.05	64.40%	*p* > 0.05
	RFCA	5	2.81	1.97–4.01	*p* < 0.01	38.70%	*p* > 0.05

CPVI, circumferential pulmonary vein isolation; CPVI plus, includes CPVI with one or more of adjuvant ablations in cavotricuspid isthmus, mitral isthmus, left atrial roof, the basal posterior wall, superior vena cava or complex fractionate atrial electrograms; AF, atrial fibrillation; CYA, cryoballoon ablation; RFCA, radiofrequency ablation.

## Discussion

The aim of this meta-analysis and systematic review was to examine whether LAAFV is a reliable predictor of AF recurrence after CA. Our meta-analysis showed that patients with AF recurrence had lower mean LAAFV values than those without recurrence. Moreover, we evaluated the association between LAAFV and the risk of AF recurrence after CA. Every 1cm/s rise in LAAFV, the risk of AF recurrence after CA decreased by 3% in the pooled analysis of continuous factors, whereas lower LAAFV was associated with an increased risk of AF recurrence in the pooled analysis of categorical variables. The subgroup analyses showed that the association between LAAFV and AF recurrence after CA was not significantly affected by the AF type and ablation procedure.

Catheter ablation is the most frequently performed interventional electrophysiological therapy for AF. Ectopic pacing sites in patients with AF usually originate from pulmonary veins (PV), and pulmonary vein isolation (PVI) is the cornerstone of CA. Although the surgical effect is satisfactory, the long-term recurrence rate post-operative remains high, because non-PV areas other than PVI may be the source of initiation and maintenance of AF (28–30). The most common areas include the superior vena cava, the coronary sinus, the ligament of marshall, the crista terminalis, the LA posterior wall and the LAA (31–33). LAA is a finger-like projection extending from the main body of the LA and is primarily formed by the adsorption of the primordial PV and their branches (34). It is demonstrated that LAA is a significant source of AF and atrial tachycardia (35). A study (29) found that nearly 30 percent of AF triggers originate from non-pulmonary veins, especially the LAA. Di Biase et al. (36) analyzed 987 patients undergoing AF cablation, demonstrating that 27% of AF patients were triggered by LAA, and LAA electrical isolation can improve the success rate of AF cablation. The function of the LAA is most commonly determined by measuring emptying velocity with pulsed-wave Doppler (15). Previous studies have reported that lower LAAFV in AF patients was associated with a higher risk of thromboembolism (37) and a lower success rate of long-term cardioversion (38, 39). LAAFV, representing a hemodynamic feature of LA and the LAA (40), is widely acknowledged as a marker of LAA function (including contractility, stunning, and fibrosis) (41, 42). AF is associated with pathological changes such as remodeling, electrical intolerance changes, and atrial mass loss, which lead to enlargement and dysfunction of LAA, thus causing a decrease in LAA blood flow and ultimately a reduction in LAAFV values (43).

Left atrial remodeling, including LA enlargement, hypertrophy, and/or fibrosis is the basis of AF recurrence (44). Moreover, LA size increases and LA voltages decrease due to LA remodeling, which are considered the surrogate for LA fibrosis and the significant predictor of AF recurrence after CA (45, 46). According to recent research, LAAFV is positively correlated with LA voltage and negatively correlated with LA volume (47, 48). As an indicator reflecting LA contractile and reserve function, LAAFV demonstrates the severity of LA functional remodeling, which may occur in the early stage of LA remodeling (49). Paroxysmal AF patients are generally in the early stage of LA remodeling. Chronic pressure overload causes LA enlargement, while Impaired LA function precedes LA expansion. Therefore, LAAFV may be a more sensitive predictor for AF recurrence than LA size and volume, particularly in patients with paroxysmal AF. LAA has a stronger contraction and extension function than the LA, the distensible at a greater degree than the LA, as buffering effect on reducing LA pressure (50). It provides a theoretical basis that flow velocity of LAA may be a more dependable parameter for AF recurrence than LA. LAAFV, which reflects the more comprehensive LAA dysfunction and atrial remodeling as mentioned above, mainly depends on the contraction of LAA, and therefore, would be a more reliable predictor of AF recurrence. The primary imaging method for assessing LAAFV is TEE (51), which provides a more accurate risk assessment because it allows the characterization of the AF substrates (52).

Although there have been many studies on the relationship between LAAFV and AF recurrence after CA, how to determine the cut-off value of LAAFV is a crucial question to be answered. In our meta-analysis, the cut-off values of the seven studies that defined LAAFV as categorical variables were displayed in Table 7. The cut-off values of most studies are similar, and the differences across studies were probably caused by different research populations or methods, and in the largest related studies so far, the cut-off value of 40 cm/s has been proposed.

**TABLE 7:** The cut-off values of the seven studies defined LAAFV as categorical variables.

Study	No. of patients	Study design	Cut-off values	OR (CI) recurrence	Sensitivity	Specificity
Gerede et al. (12)	51	prospective	<30 cm/s	1.13 (1.12–1.23)	85%	95%
Kiełbasa et al. (13)	417	retrospective	<45 cm/s	1.63 (1.06–2.49)	–	–
Fukushima et al. (15)	105	prospective	<48.5 cm/s	2.68 (1.14–6.32)	–	–
Istratoaie et al. (18)	81	prospective	<40.5 cm/s	8.59 (3.13–23.60)	89%	75%
Kanda et al. (20)	53	prospective	<28 cm/s	3.24 (1.24-8.48)	62%	69%
Yang et al. (22)	164	retrospective	<37 cm/s	2.32 (1.18–4.23)	60.5%	66.9%
Kim et al. (26)	2352	retrospective	<40 cm/s	2.35 (1.97–2.79)	–	–

To our knowledge, this is the first meta-analysis to summarize the association between the LAAFV and AF recurrence after CA. The advantages of the meta-analysis may include the following. First, the results of this study were relatively stable and reliable because the meta-analysis covered studies from different countries and had a large sample size. Second, the finding that LAAFV is associated with the risk of AF recurrence after CA was based on most adequately adjusted ORs, suggesting that the finding may not be affected by potential confounding factors. Third, studies with LAAFV analyzed as categorized and continuous data were summarized separately and derived consistent results, which further verified the stability of the results. Fourth, the sensitivity analyses by removing one individual study at a time had no significant impact on the results, suggesting the outcomes were credible. Fifth, multiple subgroup analyses were conducted to assess the potential study characteristics of the relationship between LAAFV and AF recurrence after CA.

However, this meta-analysis also had some limitations. First, as a meta-analysis of observational studies, it carries inherent limitations of the study design. Second, the heterogeneity of our study was significant. Even if sensitivity and subgroup analyses were adopted, the origin of heterogeneity could not be explored. Third, we cannot rule out that some residual factors may confuse the link between LAAFV and AF recurrence. Forth, studies that defined LAAFV as categorical variables have different cut-off values, which would impact our study result.

## Conclusion

Meta-analyses of observational studies show that patients with AF recurrence after CA have lower mean LAAFV values than patients without recurrence. Lower LAAFV was associated with an increased risk of AF recurrence after CA, and the assessment of LAAFV before CA could be used as a potential and feasible screening method to predict the risk of AF recurrence. Further studies with larger, well designed, and randomized studies with longer follow up periods for LAAFV should be conducted. In addition, the mechanism of LAAFV and AF recurrences remains to be further explored.

## Data availability statement

The original contributions presented in this study are included in the article/Supplementary material, further inquiries can be directed to the corresponding authors.

## Author contributions

JD and ZC were involved in conceptualization and supervision. PC, LM, and XG collected, analyzed, and interpreted the data. PC, YS, and DP drew pictures and wrote the original draft. JJ and JD edited and modified the final version. All authors contributed to the article and approved the submitted version.

## Supplementary material

SUPPLEMENTARY FIGURE 1 Funnel plot for the difference in LAAFV values between patients with and without AF recurrence after CA.

SUPPLEMENTARY FIGURE 2 Egger’s test for the difference in LAAFV values between patients with and without AF recurrence after CA.

SUPPLEMENTARY FIGURE 3 Sensitivity analysis for the difference in LAAFV values between patients with and without AF recurrence after CA.

SUPPLEMENTARY FIGURE 4 Sensitivity analysis for the relationship between LAAFV (continuous variables) and the risk of AF recurrence after CA.

SUPPLEMENTARY FIGURE 5 Sensitivity analysis for the relationship between LAAFV (categorical variables) and the risk of AF recurrence after CA.
