# Supplementary material for: Left atrial appendage flow velocity predicts recurrence of atrial fibrillation after catheter ablation: A systematic review and meta-analysis
Source: Front Cardiovasc Med. 2022 Sep 6;9:971848. doi: 10.3389/fcvm.2022.971848 (PMC9485569; doi:10.3389/fcvm.2022.971848)
Supplement: Supplementary file 1 [file Data_Sheet_1.pdf]

Database (Search Date)	Search Terms	Filters	Number of results
Pubmed (1/5/2022)	#1 Atrial Fibrillation[mh] OR Fibrillation*, Atrial[tiab] OR Auricular Fibrillation*[tiab] OR Persistent Atrial Fibrillation*[tiab] OR Paroxysmal Atrial Fibrillation*[tiab] OR Familial Atrial Fibrillation*[tiab]<br>#2 Catheter Ablation[mh] OR Cryosurgery[mh] OR Radiofrequency Ablation[mh] OR Ablation, Catheter[tiab] OR Transvenous Catheter Ablation[tiab] OR Electrical* Catheter Ablation[tiab] OR Transvenous Electric* Ablation[tiab] OR Percutaneous Catheter Ablation[tiab] OR Cryosurgeries[tiab] OR Cryoablation*[tiab] OR Circumferential Pulmonary Vein Isolation[tiab]<br>#3 Left Atrial Appendage Flow Velocity[tiab] OR Left Atrial Appendage Emptying Velocity[tiab]<br>#4 Recurrence*[tiab]<br>#5 #1 AND #2 AND #3 AND #4	Language: English	56
Embase (1/5/2022)	#1 (Atrial Fibrillation).exp.<br>#2 (Persistent Atrial Fibrillation).ti,ab.<br>#3 (Paroxysmal Atrial Fibrillation).ti,ab.<br>#4 (Auricular Fibrillation).ti,ab.<br>#5 #1 OR #2 OR #3 OR #4<br>#6 (Catheter Ablation).exp.<br>#7 (Cryosurgery).exp.<br>#8 (Radiofrequency Ablation).exp.<br>#9 (Transvenous Catheter Ablation).mp.<br>#10 (Electrical Catheter Ablation).mp.<br>#11 (Percutaneous Catheter Ablation).mp.<br>#12 (Cryosurgeries).mp.<br>#13 (Cryoablation).mp.<br>#14 (Circumferential Pulmonary Vein Isolation).mp.<br>#15 #6 OR #7 OR #8 OR #9 OR #10 OR #11 OR #12 OR #13 OR #14<br>#16 (Left Atrial Appendage Flow Velocity).mp.<br>#17 (Left Atrial Appendage Emptying Velocity).mp.<br>#18 #16 OR #17<br>#19 (Recurrence).mp.<br>#20 #5 AND #15 AND #18 AND #19	Language: English	124
Web of science	#1 Topic:(Atrial Fibrillation OR Auricular Fibrillation OR Persistent Atrial Fibrillation OR Paroxysmal Atrial	Language:	74

(1/5/2022)	Fibrillation)<br>Databases=SCI-EXPANDED, SSCI, A&HCI, CPCI-S, CPCI-SSH, BKCI-S, BKCI-SSH, ESCI, CCR-EXPANDED, IC Timespan= 1970-2022<br>#2 Topic:(Catheter Ablation OR Cryosurgery OR Radiofrequency Ablation OR Transvenous Catheter Ablation OR Electrical* Catheter Ablation OR Percutaneous Catheter Ablation OR Cryosurgeries OR Cryoablation) OR Circumferential Pulmonary Vein Isolation)<br>Databases=SCI-EXPANDED, SSCI, A&HCI, CPCI-S, CPCI-SSH, BKCI-S, BKCI-SSH, ESCI, CCR-EXPANDED, IC Timespan= 1970-2022<br>#3 Topic:(Left Atrial Appendage Flow Velocity) OR (Left Atrial Appendage Emptying Velocity)<br>Databases=SCI-EXPANDED, SSCI, A&HCI, CPCI-S, CPCI-SSH, BKCI-S, BKCI-SSH, ESCI, CCR-EXPANDED, IC Timespan= 1970-2022<br>#4 Topic:(Recurrence*)<br>Databases=SCI-EXPANDED, SSCI, A&HCI, CPCI-S, CPCI-SSH, BKCI-S, BKCI-SSH, ESCI, CCR-EXPANDED, IC Timespan= 1970-2022<br>#5 #1 AND #2 AND #3 AND #4	English	
Cochrane Library (1/5/2022)	AF OR “Atrial Fibrillation” OR “Auricular Fibrillation*” OR “Persistent Atrial Fibrillation*” OR “Paroxysmal Atrial Fibrillation*” OR “Familial Atrial Fibrillation*” in Title Abstract Keyword AND “Catheter Ablation” OR “Cryosurgery” OR “Radiofrequency Ablation” OR “Transvenous Catheter Ablation” OR “Electrical* Catheter Ablation” OR “Transvenous Electric* Ablation” OR “Percutaneous Catheter Ablation” OR Cryosurgeries* OR Cryoablation* OR “Circumferential Pulmonary Vein Isolation” in Title Abstract Keyword AND “Left Atrial Appendage Flow Velocity” OR “Left Atrial Appendage Emptying Velocity” OR “Recurrence*” in Title Abstract Keyword - (Word variations have been searched)	Language: English	8
Scopus (1/5/2022)	(TITLE-ABS-KEY (“Atrial Fibrillation*”) OR TITLE-ABS-KEY (“Auricular Fibrillation*”) OR TITLE-ABS-KEY (“Persistent Atrial Fibrillation*”) OR TITLE-ABS-KEY (“Paroxysmal Atrial Fibrillation*”) OR TITLE-ABS-KEY (“Familial Atrial Fibrillation*”) OR TITLE-ABS-KEY (AF*)) AND (TITLE-ABS-KEY (“Catheter Ablation*”) OR TITLE-ABS-KEY (Cryosurgery*) OR TITLE-ABS-KEY ("Radiofrequency	Language: English	54

	Ablation*") OR TITLE-ABS-KEY ("Transvenous Catheter Ablation*") OR TITLE-ABS-KEY ("Electrical* Catheter Ablation") OR TITLE-ABS-KEY ("Transvenous Electric* Ablation") OR TITLE-ABS-KEY ("Percutaneous Catheter Ablation") OR TITLE-ABS-KEY (Cryosurgeries*) OR TITLE-ABS-KEY ("Circumferential Pulmonary Vein Isolation")) AND (TITLE-ABS-KEY ("Left Atrial Appendage Flow Velocity") OR TITLE-ABS-KEY ("Left Atrial Appendage Emptying Velocity")) AND (TITLE-ABS-KEY (Recurrence*))		
CINAHL (1/5/2022)	((MH "Atrial Fibrillation") OR (MH "Auricular Fibrillation") OR (MH "Persistent Atrial Fibrillation") OR (MH "Paroxysmal Atrial Fibrillation") OR (MH "Familial Atrial Fibrillation") OR (MH "AF"))<br>AND<br>((MH "Catheter Ablation") OR (MH "Cryosurgery") OR (MH "Radiofrequency Ablation") OR (MH "Transvenous Catheter Ablation") OR (MH "Electrical Catheter Ablation") OR (MH "Transvenous Electric Ablation") OR (MH "Percutaneous Catheter Ablation") OR (MH "Cryosurgeries") OR (MH "Circumferential Pulmonary Vein Isolation"))<br>AND<br>TX ("Left Atrial Appendage Flow Velocity" OR "Left Atrial Appendage Emptying Velocity" OR "Recurrence")	Language: English	6

Full Search Strategy and Results

The Exclusion Studies and Reason

Reason for Exclusion	Study Name	Reference
not report the AF recurrence outcomes	Chen et al 2021	Chen L, Xu C, Chen W, Zhang C. Left atrial appendage orifice area and morphology is closely associated with flow velocity in patients with nonvalvular atrial fibrillation. BMC Cardiovasc Disord. 2021 Sep 16;21(1):442.

not report the AF recurrence outcomes	Xu et al 2021	Xu B, Du Y, Xu C, Sun Y, Peng F, Wang S, Pan J, Lou Y, Xing Y. Left Atrial Appendage Morphology and Local Thrombogenesis-Related Blood Parameters in Patients With Atrial Fibrillation. J Am Heart Assoc. 2021 Jun 15;10(12):e020406.
not report the AF recurrence outcomes	Gawałko et al 2020	Gawałko M, Budnik M, Uziębło-Życzkowska B, Krzesiński P, Scisło P, Kochanowski J, Jurek A, Kiliszek M, Gielerak G, Filipiak KJ, Opolski G, Kapłon-Cieślicka A. Decreased left atrial appendage emptying velocity as a link between atrial fibrillation type, heart failure and older age and the risk of left atrial thrombus in atrial fibrillation. Int J Clin Pract. 2020 Nov;74(11):e13609.
not report the AF recurrence outcomes	Fiala M et al 2014	Fiala M, Wichterle D, Bulková V, Sknouril L, Nevralová R, Toman O, Dorda M, Januska J, Spinar J. A prospective evaluation of haemodynamics, functional status, and quality of life after radiofrequency catheter ablation of long-standing persistent atrial fibrillation. Europace. 2014 Jan;16(1):15-25.
not report the AF recurrence outcomes	Kim et al 2021	Kim YG, Min K, Hwang SH, Shim J, Choi YY, Choi HY, Choi JI, Oh YW, Kim YH. Blood flow volume of left atrial appendage measured by magnetic resonance imaging is improved after radiofrequency catheter ablation of atrial fibrillation. J Cardiovasc Electrophysiol. 2021 Mar;32(3):669-677.
not report the AF recurrence outcomes	Kiedrowicz et al 2021	Kiedrowicz RM, Wielusinski M, Wojtarowicz A, Kazmierczak J. Left and right atrial appendage functional features as predictors for voltage-defined left atrial remodelling in patients with long-standing persistent atrial fibrillation. Heart Vessels. 2021 Jun;36(6):853-862.

not report the AF recurrence outcomes	Combes et al 2013	Combes S, Jacob S, Combes N, Karam N, Chaumeil A, Guy-Moyat B, Treguer F, Deplagne A, Boveda S, Marijon E, Albenque JP. Predicting favourable outcomes in the setting of radiofrequency catheter ablation of long-standing persistent atrial fibrillation: a pilot study assessing the value of left atrial appendage peak flow velocity. Arch Cardiovasc Dis. 2013 Jan;106(1):36-43.
not report the AF recurrence outcomes	Machino-Ohtsuka et al 2013	Machino-Ohtsuka T, Seo Y, Ishizu T, Yanaka S, Nakajima H, Atsumi A, Yamamoto M, Kawamura R, Koshino Y, Machino T, Kuroki K, Yamasaki H, Igarashi M, Sekiguchi Y, Tada H, Aonuma K. Significant improvement of left atrial and left atrial appendage function after catheter ablation for persistent atrial fibrillation. Circ J. 2013;77(7):1695-704.
not report the AF recurrence outcomes	Kusa et al 2015	Kusa S, Komatsu Y, Taniguchi H, Uchiyama T, Takagi T, Nakamura H, Miyazaki S, Hachiya H, Iesaka Y. Left atrial appendage flow velocity after successful ablation of persistent atrial fibrillation: clinical perspective from transesophageal echocardiographic assessment during sinus rhythm. Am Heart J. 2015 Feb;169(2):211-21.
not report the AF recurrence outcomes	Antonielli et al 2002	Antonielli E, Pizzuti A, Pálinkás A, Tanga M, Gruber N, Michelassi C, Varga A, Bonzano A, Gandolfo N, Halmai L, Bassignana A, Imran MB, Delnevo F, Csanády M, Picano E. Clinical value of left atrial appendage flow for prediction of long-term sinus rhythm maintenance in patients with nonvalvular atrial fibrillation. J Am Coll Cardiol. 2002 May 1;39(9):1443-9.
not report the LAAFV values	Kim et al 2018	Kim YG, Shim J, Oh SK, Park HS, Lee KN, Hwang SH, Choi JI, Kim YH. Different Responses of Left Atrium and Left Atrial Appendage to Radiofrequency Catheter Ablation of Atrial Fibrillation: a Follow Up MRI study. Sci Rep. 2018 May 18;8(1):7871.

not report the LAAFV values	Krul et al 2015	Krul SP, Berger WR, Smit NW, van Amersfoorth SC, Driessen AH, van Boven WJ, Fiolet JW, van Ginneken AC, van der Wal AC, de Bakker JM, Coronel R, de Groot JR. Atrial fibrosis and conduction slowing in the left atrial appendage of patients undergoing thoracoscopic surgical pulmonary vein isolation for atrial fibrillation. Circ Arrhythm Electrophysiol. 2015 Apr;8(2):288-95.
not report the LAAFV values	Chilukuri et al 2010	Chilukuri K, Mayer SA, Scherr D, Dalal D, Abraham T, Henrikson CA, Cheng A, Nazarian S, Sinha S, Spragg D, Berger R, Calkins H, Marine JE. Transoesophageal echocardiography predictors of periprocedural cerebrovascular accident in patients undergoing catheter ablation of atrial fibrillation. Europace. 2010 Nov;12(11):1543-9.
not report the LAAFV values	Li et al 2022	Li Y, Han B, Li J, Ge F, Yang L. Value of echocardiography in evaluating efficacy of radiofrequency catheter ablation in patients with atrial fibrillation. Am J Transl Res. 2022 Mar 15;14(3):1778-1787.
not use CA as an intervention	Fukuhara et al 2021	Fukuhara E, Mine T, Kishima H, Ishihara M. Predictors for reduced flow velocity in left atrial appendage during sinus rhythm in patients with atrial fibrillation. Heart Vessels. 2021 Mar;36(3):393-400.
not use CA as an intervention	Harada et al 2018	Harada M, Koshikawa M, Motoike Y, Ichikawa T, Sugimoto K, Watanabe E, Ozaki Y. Left Atrial Appendage Thrombus Prior to Atrial Fibrillation Ablation in the Era of Direct Oral Anticoagulants. Circ J. 2018 Oct 25;82(11):2715-2721.
not use CA as an intervention	Wang et al 2005	Wang YC, Lin LC, Lin MS, Lai LP, Hwang JJ, Tseng YZ, Tseng CD, Lin JL. Identification of good responders to rhythm control of paroxysmal and persistent atrial fibrillation by transthoracic and transesophageal echocardiography. Cardiology. 2005;104(4):202-9.

Review	Kornej et al 2014	Kornej J, Husser D, Bollmann A, Lip GY. Rhythm outcomes after catheter ablation of atrial fibrillation. Clinical implication of biomarkers. Hamostaseologie. 2014;34(1):9-19.
Review	Nishimura et al 2019	Nishimura M, Lupercio-Lopez F, Hsu JC. Left Atrial Appendage Electrical Isolation as a Target in Atrial Fibrillation. JACC Clin Electrophysiol. 2019 Apr;5(4):407-416.
