# Supplementary figures and images for: Left atrial appendage flow velocity predicts recurrence of atrial fibrillation after catheter ablation: A systematic review and meta-analysis
Source: Front Cardiovasc Med. 2022 Sep 6;9:971848. doi: 10.3389/fcvm.2022.971848 (PMC9485569; doi:10.3389/fcvm.2022.971848)

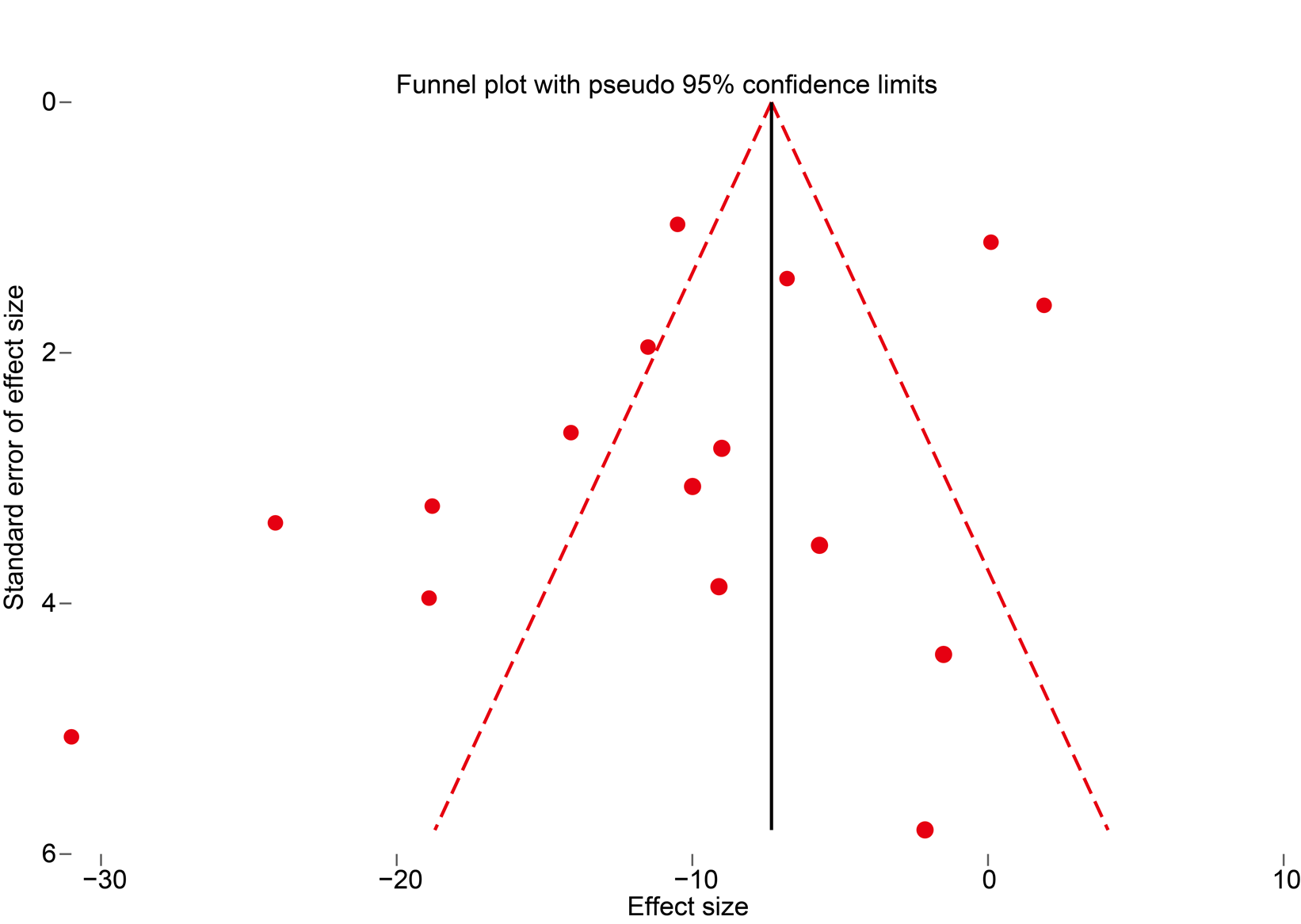

Supplement: SUPPLEMENTARY FIGURE 1 — Funnel plot for the difference in LAAFV values between patients with and without AF recurrence after CA. [file Image_1.tif]

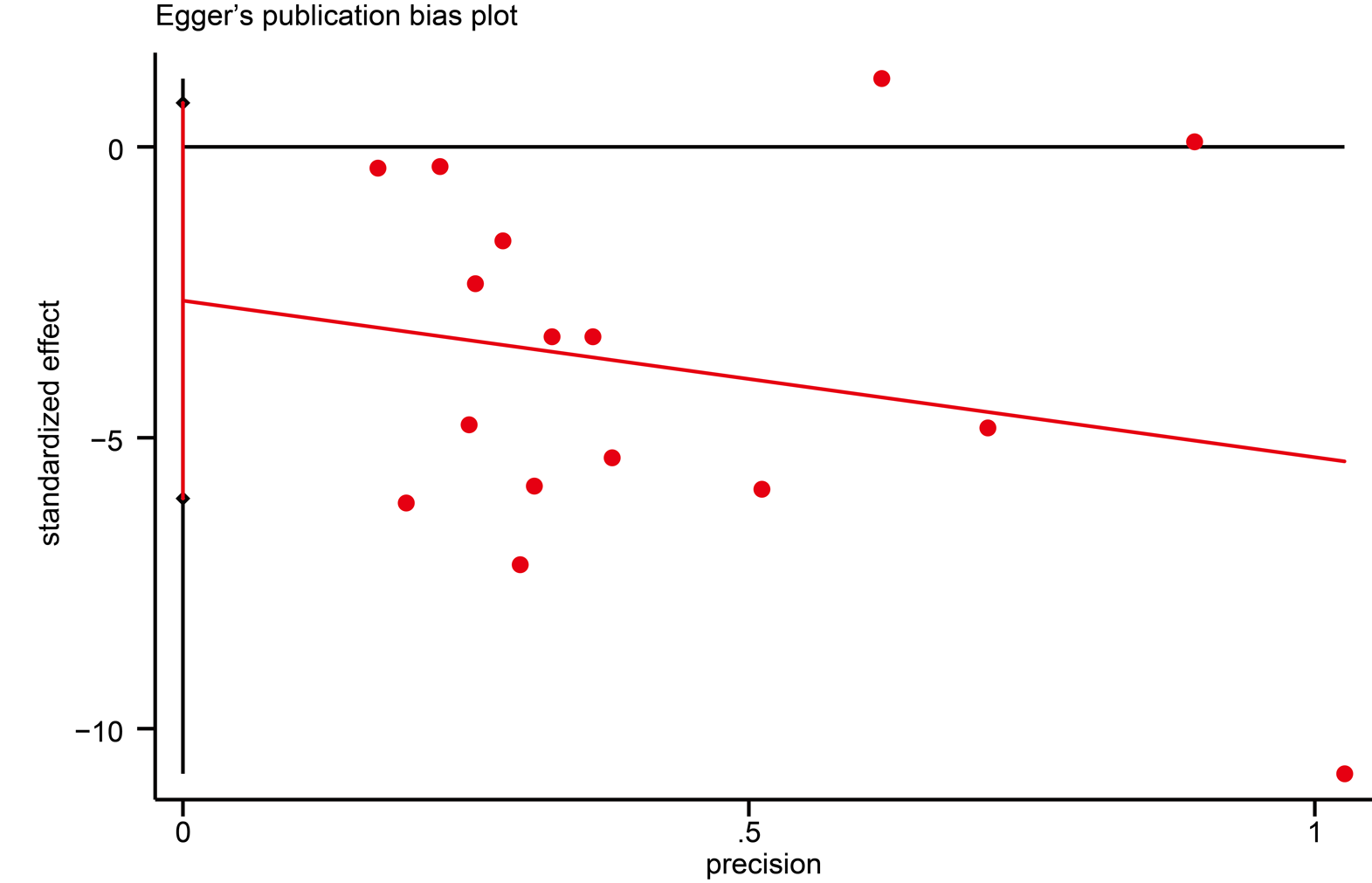

Supplement: SUPPLEMENTARY FIGURE 2 — Egger’s test for the difference in LAAFV values between patients with and without AF recurrence after CA. [file Image_2.tif]

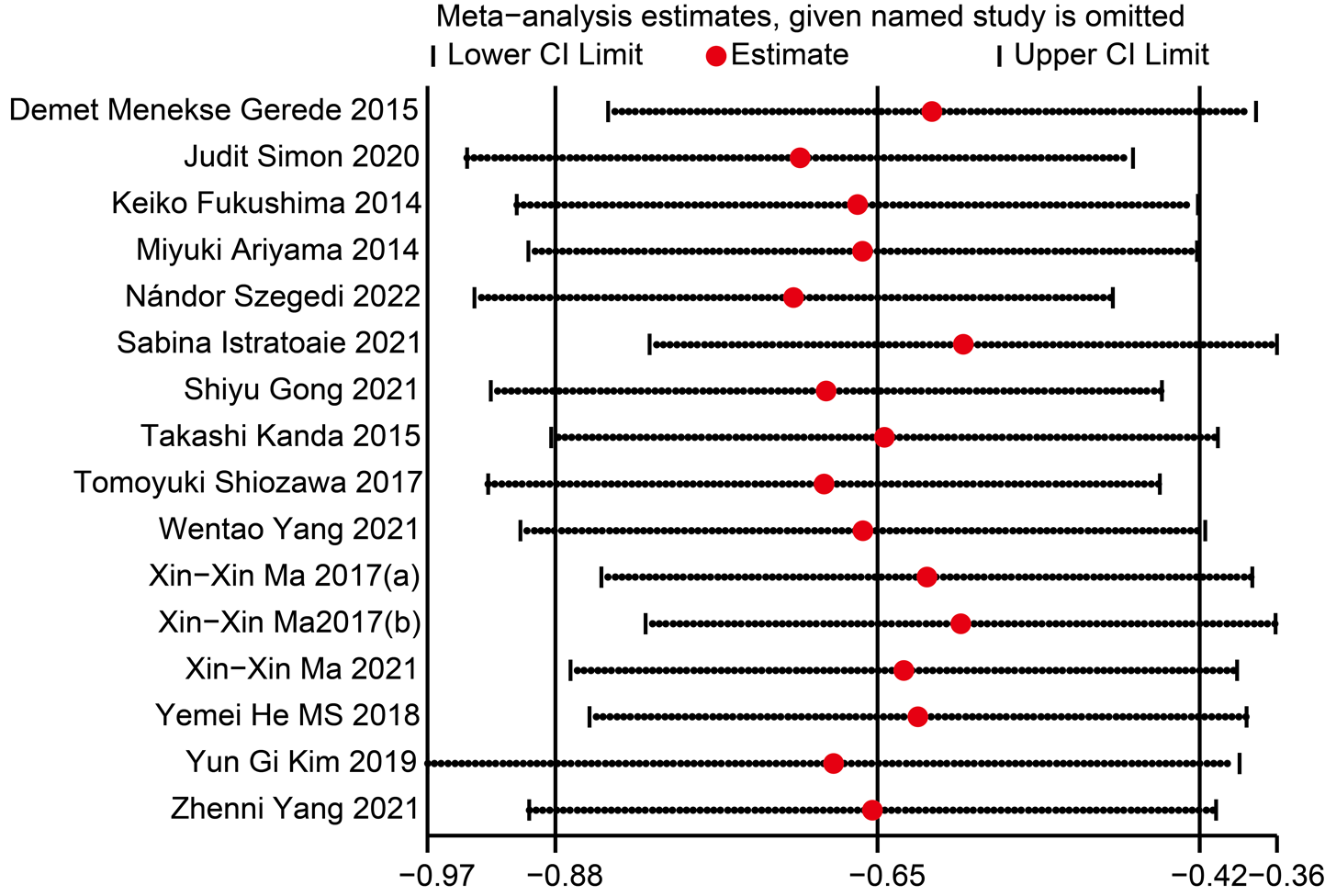

Supplement: SUPPLEMENTARY FIGURE 3 — Sensitivity analysis for the difference in LAAFV values between patients with and without AF recurrence after CA. [file Image_3.tif]

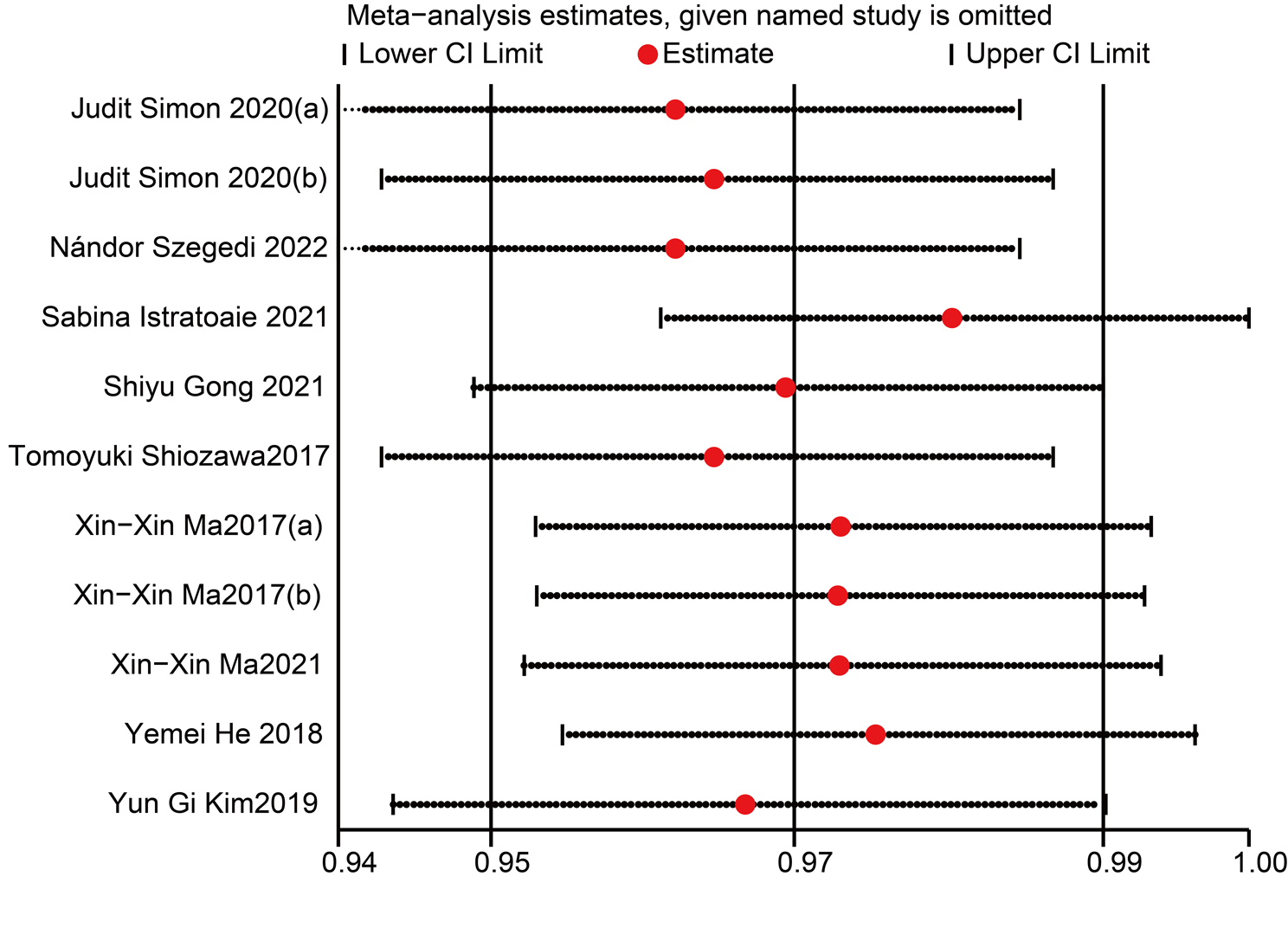

Supplement: SUPPLEMENTARY FIGURE 4 — Sensitivity analysis for the relationship between LAAFV (continuous variables) and the risk of AF recurrence after CA. [file Image_4.tif]

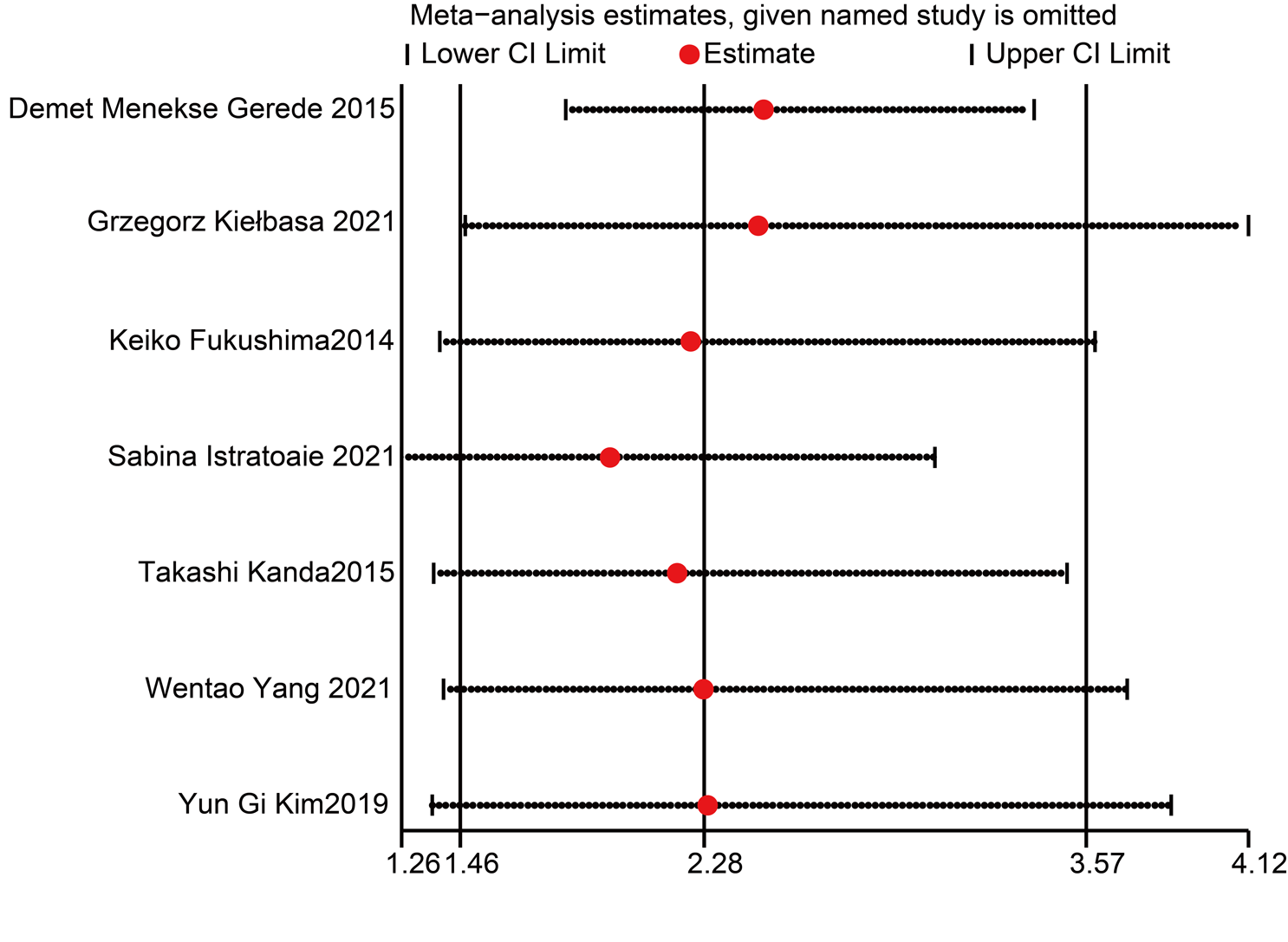

Supplement: SUPPLEMENTARY FIGURE 5 — Sensitivity analysis for the relationship between LAAFV (categorical variables) and the risk of AF recurrence after CA. [file Image_5.tif]
